# Angiotensin blockade therapy and survival in pancreatic cancer: a population study

**DOI:** 10.1186/s12885-022-09200-4

**Published:** 2022-02-07

**Authors:** Scott W. Keith, Vittorio Maio, Hwyda A. Arafat, Matthew Alcusky, Thomas Karagiannis, Carol Rabinowitz, Harish Lavu, Daniel Z. Louis

**Affiliations:** 1 Division of Biostatistics, Department of Pharmacology and Experimental Therapeutics, Sidney Kimmel Medical College, Thomas Jefferson University, 130 S 9th St., 17th Floor, 19107 Philadelphia, PA USA; 2 Jefferson College of Population Health, Thomas Jefferson University, 901 Walnut Street, 10th Floor, 19107 Philadelphia, PA USA; 3 Asano-Gonnella Center for Research in Medical Education and Health Care, Sidney Kimmel Medical College, Thomas Jefferson University, 1015 Walnut Street, Suite 319, 19107 Philadelphia, PA USA; 4 Department of Biomedical Sciences, University of New England, 11 Hills Beach Road, 04005 Biddeford, Maine USA; 5 Division of Epidemiology, Department of Population and Quantitative Health Sciences, University of Massachusetts Medical School, Worcester, MA USA; 6 Department of Surgery, Sidney Kimmel Medical College, Thomas Jefferson University, 1025 Walnut St., College Bldg., 6th Floor, 19107 Philadelphia, PA USA

**Keywords:** Angiotensin I converting enzyme (ACE) inhibitors, Angiotensin receptor blockers (ARBs), Mortality, Pancreatic cancer, Pharmacoepidemiology, Survival

## Abstract

**Background:**

Pancreatic cancer (PC) is one of the most aggressive and challenging cancer types to effectively treat, ranking as the fourth-leading cause of cancer death in the United States. We investigated if exposures to angiotensin II receptor blockers (ARBs) or angiotensin I converting enzyme (ACE) inhibitors after PC diagnosis are associated with survival.

**Methods:**

PC patients were identified by ICD-9 diagnosis and procedure codes among the 3.7 million adults living in the Emilia-Romagna Region from their administrative health care database containing patient data on demographics, hospital discharges, all-cause mortality, and outpatient pharmacy prescriptions. Cox modeling estimated covariate-adjusted mortality hazard ratios for time-dependent ARB and ACE inhibitor exposures after PC diagnosis.

**Results:**

8,158 incident PC patients were identified between 2003 and 2011, among whom 20% had pancreas resection surgery, 36% were diagnosed with metastatic disease, and 7,027 (86%) died by December 2012. Compared to otherwise similar patients, those exposed to ARBs after PC diagnosis experienced 20% lower mortality risk (HR=0.80; 95% CI: 0.72, 0.89). Those exposed to ACE inhibitors during the first three years of survival after PC diagnosis experienced 13% lower mortality risk (HR=0.87; 95% CI: 0.80, 0.94) which attenuated after surviving three years (HR=1.14; 95% CI: 0.90, 1.45).

**Conclusions:**

The results of this large population study suggest that exposures to ARBs and ACE inhibitors after PC diagnosis are significantly associated with improved survival. ARBs and ACE inhibitors could be important considerations for treating PC patients, particularly those with the worst prognosis and most limited treatment options. Considering that these common FDA approved drugs are inexpensive to payers and present minimal increased risk of adverse events to patients, there is an urgent need for randomized clinical trials, large simple randomized trials, or pragmatic clinical trials to formally and broadly evaluate the effects of ARBs and ACE inhibitors on survival in PC patients.

**Supplementary Information:**

The online version contains supplementary material available at 10.1186/s12885-022-09200-4.

## Background

Pancreatic cancer (PC) is among the most aggressive forms of cancer with an estimated incidence of 60,430 and causing 48,220 deaths in 2021, ranking it as the fourth-leading cause of cancer death in the United States [1]. Currently, surgical resection remains the only treatment option consistently achieving significantly prolonged survival [2], but due to the characteristically late presentation of the disease, only 10 – 20% of patients are candidates for resection [3]. Despite the significant research devoted to PC and its treatment in those with unresectable disease, there have been only modest improvements in overall survival relative to other common malignancies over the last two decades  [3].

Extensive preclinical data support the potential use of angiotensin II receptor blockers (ARBs) and angiotensin I converting enzyme (ACE) inhibitors, two common classes of medications FDA approved for the management of hypertension [4], as antineoplastic agents in PC. In particular, ARBs have been shown to exert multiple effects on PC cell metabolism and cell cycle machinery [4–7]. An early retrospective cohort study first introduced the potential association between angiotensin inhibition and lower cancer incidence [8]. Subsequent studies used a variety of study designs and failed to establish a link between these agents and reduced cancer incidence [9, 10]. Following more recent positive preclinical data, multiple studies have also explored the association between angiotensin inhibition and overall survival in different types of cancer. For example, both ARBs and ACE inhibitors were shown to be associated with an increase in median overall survival in patients with non-squamous cell lung cancer and patients with gastric cancer [11, 12]. Similar survival benefits from angiotensin inhibition also appear in PC. One small single-center retrospective study found that ARB or ACE inhibitor use was associated with an increase in median overall survival in patients with advanced PC receiving gemcitabine monotherapy (15.1 months vs. 8.9 months) [13]. Another found that, among patients who survived at least 6 months, ARB prescriptions were associated with 24% lower mortality in PC patients undergoing surgery for pancreas resection [14]. Two small phase II clinical trials have been initiated to investigate if including an ARB (losartan) in the treatment of non-metastatic PC patients might improve success in surgical tumor resection [15, 16]. Some recent findings suggest a possible benefit among locally advanced patients [15].

To our knowledge, there are no large population-based studies evaluating the associations of exposures to ARBs and ACE inhibitors with mortality outcomes in a general population of PC patients. We retrospectively evaluated a large population-based cohort of patients diagnosed with PC to investigate the relationships between overall mortality and exposures to ARBs and ACE inhibitors after PC diagnosis.

## Methods

### Setting and study data

Italy’s National Health Service provides universal health care coverage to all citizens. The data for this study were drawn from the longitudinal health care database of the approximately 3.7 million adults in the population served by the Regional Health Service System of Emilia-Romagna, a northern Italian region of approximately 4.5 million inhabitants, between January 1, 2002 and December 31, 2011 [17]. The database provided administrative linkable de-identified patient information records on demographics, hospital discharge data (utilizations characterized by ICD-9-CM diagnosis and procedure codes, as well as admission and discharge dates), all-cause mortality (mortality status and date of either death or censoring for moving out of the region or censoring for surviving to the end of follow-up) and outpatient pharmacy data on individual prescriptions (Anatomical Therapeutic Chemical codes identifying which drugs were prescribed). This database has been used extensively for pharmacoepidemiologic research [18–20].

### Case status, metastasis, and resection identification

Cases were those having hospital discharge records with ICD-9-CM diagnosis codes indicating a primary or secondary diagnosis for malignant neoplasm of the pancreas (i.e., 157.X; see Supplemental Table S1 for details). Lymph node involvement and metastases were indicated by secondary malignancies at other sites (i.e., 196.X, 197.X, 198.X, 199.X; see Supplemental Table S2 for details). Pancreatic resection was indicated by presence of pancreatectomy ICD-9 procedure codes (i.e., 52.XX; see Supplemental Table S3 for details).

### Drug exposures, chemotherapy, and radiotherapy

We tracked exposure to ARBs and ACE inhibitors, as well as exposure to drug classes potentially impacting cancer progression or pertinent diseases, including aspirin, alpha blockers, beta blockers, calcium channel blockers, diuretics, non-steroidal anti-inflammatory drugs (NSAIDs), statins, metformin, and other diabetes medications. The outpatient pharmacy records available indicated only what drug was prescribed and the date it was filled. We could not determine what quantity was dispensed or the prescription duration.

Since we were tracking exposure to many drugs and fitting computationally intensive models accounting for the timing of exposures in relation to survival, the drug exposures were tracked by parsing each patient’s survival time into quarter-year periods, going forward from the time of PC diagnosis to the time of death or censoring, as well as backward 1 year, while noting in each quarter what drug classes had been prescribed and filled in the prior quarter. After PC diagnosis, a patient was considered exposed to a given drug class only after the first quarter in which they filled at least one prescription for a drug in that class. That is, exposure initiation was lagged to the beginning of the next quarter and once a patient had incurred exposure to a given drug class following their PC diagnosis, they were considered exposed to that drug class for the remainder of their survival follow-up. To avoid immortal time bias [21], these drug exposure indicator variables were constructed as time-dependent covariates allowed to change from unexposed to exposed for any PC patient. Time dependent covariates were similarly constructed for chemotherapy, radiotherapy, and pancreatic resection treatment status.

### Statistical analysis and mortality modeling

The distributions of sample characteristics were summarized by medians with the first and third quartiles if continuous and by frequency counts and percentages if categorical. In addition to time-dependent ARB and ACE inhibitor exposure indicator variables, potential confounders summarized and used in modeling included demographic variables (age, age squared, and sex), a geography variable (indicating if a patient’s residence lies in a plain, in the hills, or in the mountains, which serves as a proxy measure of population density in the region), calendar time at diagnosis (quantified as years from 1/1/2002), chemotherapy, radiotherapy, pancreatic resection, metastasis at diagnosis, limitations to functional status as indicated by home health care or oxygen prescriptions in the year prior to diagnosis and/or discharge to a nursing home, the Elixhauser comorbidity measure [22] for administrative data which sums 30 comorbidity indicators, some of the most prevalent Elixhauser comorbidity indicators in these patients, exposure indicators for each of the other nine drug classes in the year prior to PC diagnosis, and time-dependent exposure indicators for those other nine drug classes after PC diagnosis. Mortality follow-up ended on December 31, 2012. The cause of death was not available.

The counting process style of input was used to construct a Cox proportional hazards all-cause mortality model of the time-dependent ARB and ACE inhibitor drug exposure variables and other time-dependent or time-fixed covariates. We have used this methodology to address a similar question on metformin exposure and survival in patients with head and neck cancers [20] Supplemental Table S4 illustrates the counting process data structure of two hypothetical PC patients showing their diagnosis dates, last follow-up date, quarterly start/stop follow-up time intervals, vital status, quarter index (looking back 5 quarters prior to PC diagnosis and forward until end of follow-up for prescriptions), ARB and ACE inhibitor prescription indicators in each quarter, and lagged time-dependent ARB and ACE exposure variables.

Models were also fitted to evaluate the mortality hazard associated with exposures to ARBs or to ACE inhibitors in subgroups of metastatic patients, pancreas resection patients, non-metastatic and non-resected patients, patients with and without, respectively, ACE inhibitor or ARB exposures in the year prior to PC diagnosis, and patients having at least one comorbidity at PC diagnosis. We detected a departure from the proportional hazards assumption for ACE inhibitors, but not for ARBs (see Supplemental Figure S1). This was addressed in each Cox model by using time-dependent coefficients for ACE inhibitor exposure, allowing its hazard ratio to change during survivorship, while the ARB coefficient was constant over time.

All statistical data analyses were conducted by Scott W. Keith, Ph.D. with SAS version 9.4 (SAS Institute, Cary, NC, USA).

## Results

We identified 8,158 PC patients diagnosed between January 2003 and December 2011 and followed them for all-cause mortality through December 2012 (Table 1). Their median age was 74.4 years and approximately half were female (51.1%). Over a third of the patients had metastatic disease at diagnosis and 20% underwent surgery for pancreatic resection. Though 19.3% of the patients had diabetes mellitus coded at diagnosis, comorbidities were not particularly common in this population as more than 75% of the sample had no more than one comorbid condition recorded and more than half had none. In the year prior to diagnosis, 18.0% of the patients were exposed to ARBs, 31.5% to ACE inhibitors, and 53.7% to neither. Over a median follow-up of 6.2 months, a total of 7,027 (86.1%) patients died, with a median survival of 6.4 months (95% CI: 6.1, 6.6). At some point following PC diagnosis, 18.0% and 31.8% were exposed to ARBs and ACE inhibitors, respectively, each for a median of 2 quarters (Supplemental Table S5).

**Table 1:** Pancreatic cancer patient characteristics (N = 8,158)

Characteristic	No.	%
**Demographics**		
Age at PC diagnosis, years		
Median [1st, 3rd quartiles]	74.4 [66.3, 81.5]	
Sex (female)	4,172	51.1
Geography		
Hill	2,290	28.1
Mountain	444	5.4
Plain	5,424	66.5
Year of PC diagnosis		
2003-2005	2,455	30.1
2006-2008	2,730	33.5
2009-2011	2,973	36.4
**Pancreatic Cancer Related Variables**		
Metastatic	2,955	36.2
Pancreas resection	1,613	19.8
Chemotherapy	3,290	40.3
Radiotherapy	855	10.5
**Comorbidities at PC diagnosis**		
Elixhauser comorbidities count 22		
Median [1st, 3rd quartiles, max]	0 [0, 1, 7]	
At Least 1 Comorbidity	3,243	39.8
Most Prevalent Elixhauser Comorbidities		
Diabetes Mellitus	1,576	19.3
Chronic Pulmonary Disease	499	6.1
Congestive Heart Failure	316	3.9
Liver Disease	262	3.2
Deficiency Anemias	259	3.2
Peripheral Vascular Disease	233	2.9
Valvular Disease	188	2.3
Neurological Disorders	181	2.2
Hypothyroidism	164	2.0
Pulmonary Circulation Disease	147	1.8
Depression	129	1.6
Chronic Blood Loss	114	1.4
**Functional Status at PC diagnosis**		
Home health care or O_2_ prescription in year prior to PC diagnosis	743	9.1
Discharged to nursing home	576	7.1
**PC patients with Drug Rx exposures in year prior to PC diagnosis**		
ACE inhibitors	2,571	31.5
ARBs	1,467	18.0
Alpha blockers	384	4.7
Beta blockers	1,695	20.8
Calcium channel blockers	1,740	21.3
Diuretics	1,676	20.5
Aspirin	2,122	26.0
NSAIDs	209	2.6
Statins	1,417	17.4
Metformin	992	12.2
Other diabetes medications	1,110	13.6
Non-users of ACEs and ARBs	4,377	53.7
Other Antihypertensive Rx	1,440	17.7
No Other Antihypertensive Rx	2,937	36.0
None of the above medications	2,318	28.4
**Time from PC diagnosis to end of follow-up**, months		
Median [1st, 3rd quartiles]	6.2 [2.4, 16.0]	
**All-cause mortality**	7,027	86.1

Abbreviations: angiotensin I converting enzyme (ACE); angiotensin II receptor blocker (ARB); hypertension prescriptions (HTN Rx); pancreatic cancer (PC); prescription (Rx)

### Time-dependent ARB and ACE inhibitor exposure indicators predicting reduced mortality

**Fig. 1:**
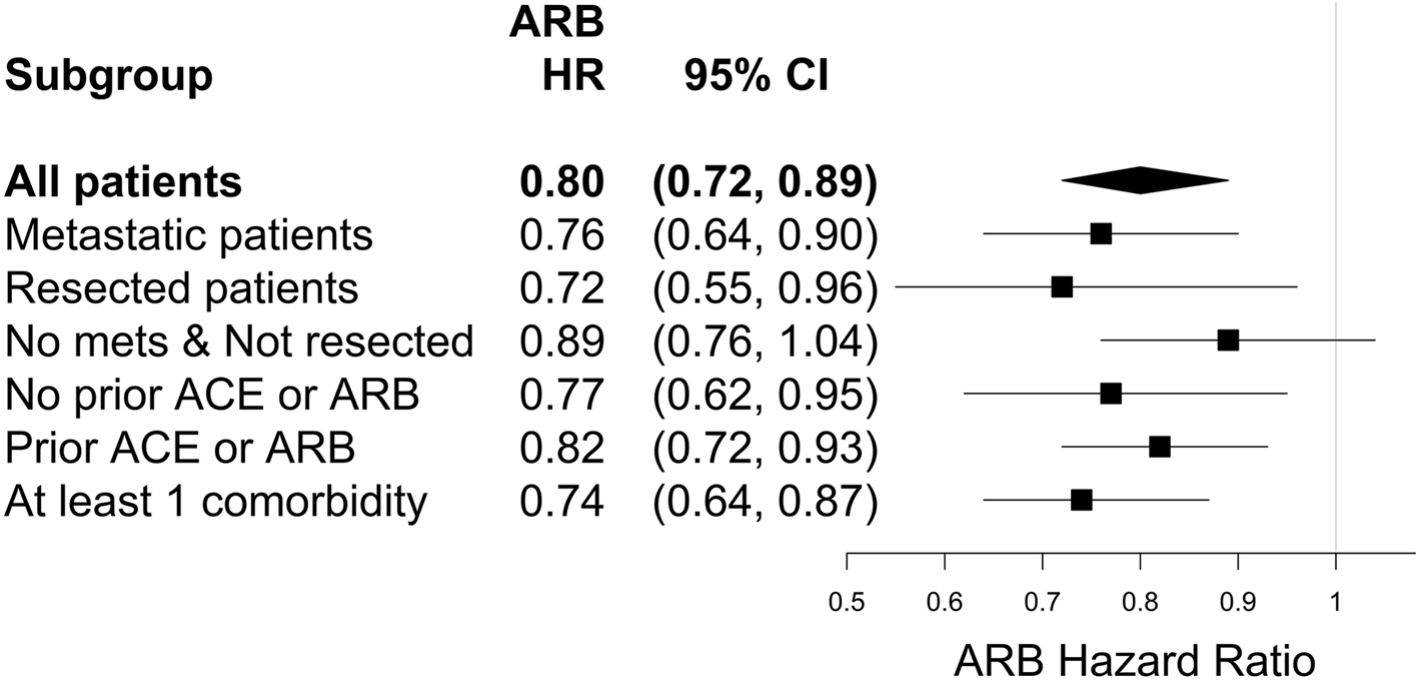
Mortality hazard ratios and 95% confidence intervals for ARB exposure after pancreatic cancer diagnosis

**Fig. 2:**
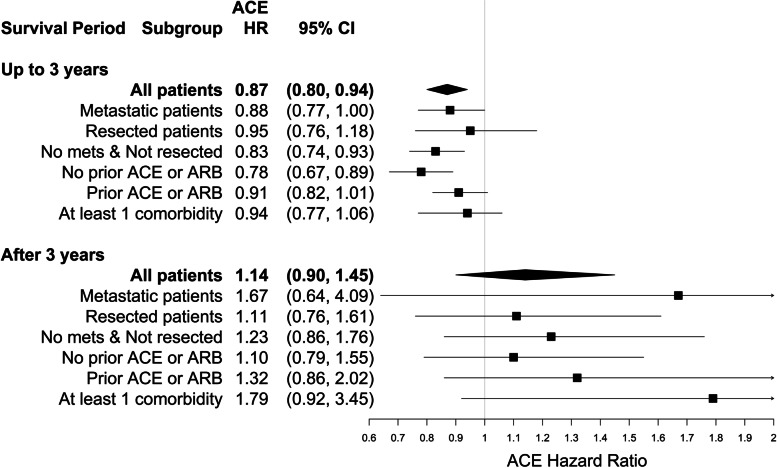
Mortality hazard ratios and 95% confidence intervals for ACE inhibitor exposure after pancreatic cancer diagnosis. This risk relationship depends on how long the patient has lived after diagnosis

Preliminary assessment of the proportional hazards assumption revealed that the association between mortality hazard and ARBs exposure after PC diagnosis was reasonably constant during survival (Supplemental Figure S1A). However, mortality hazard associated with ACE inhibitors after PC diagnosis changed after approximately 3 years of survival (Supplemental Figure S1B). Based on a Cox model adjusted for potential confounders (Supplemental Table S6), in comparison to otherwise similar patients, those exposed to ARBs after their PC diagnosis experienced 20% lower mortality hazard (Fig. 1; HR = 0.80; 95% CI: 0.72, 0.89) and those exposed to ACE inhibitors during the first three years of survival after PC diagnosis experienced 13% lower mortality hazard (Fig. 2; HR = 0.87; 95% CI: 0.80, 0.94).

However, unlike for ARBs, the reduced mortality risk associated with ACE inhibitors was no longer evident after three years of survival (Fig. 2; HR = 1.14; 95% CI: 0.90, 1.45).

### Subgroup analyses

The mortality hazards associated with ARBs and ACE inhibitors after diagnosis in particular subgroups of PC patients are also shown in Figs. 1 and 2, respectively. These results stem from independent Cox models – one fitted for each subgroup including those with metastatic disease at diagnosis, pancreas resection, no metastases at diagnosis and no pancreas resection, those with and those without ARB or ACE inhibitor exposure in the year prior to diagnosis, and those with at least one comorbidity. Significant survival benefits were associated with exposure to ARBs after PC diagnosis in each subgroup, except among the subset of non-metastatic non-pancreas resection patients. Exposure to ARBs after PC diagnosis was associated with a 28% reduction in mortality risk among resected patients (HR = 0.72; 95% CI: 0.55, 0.96), a 24% reduction in mortality risk among PC patients with metastatic disease (HR = 0.76; 95% CI: 0.64, 0.90), and a 26% reduction in mortality risk among PC patients with at least one comorbidity (HR = 0.74; 95% CI: 0.64, 0.87). The mortality hazard benefit associated with exposure to ACE inhibitors during the first three years of survival after PC diagnosis appears to be largely driven by the subgroups of patients with no metastatic disease at diagnosis and not resected (HR = 0.83; 95% CI: 0.74, 0.93) and/or those with no ARB or ACE inhibitor exposures in the year prior to their PC diagnosis (HR = 0.78; 95% CI: 0.67, 0.89). When the subgroup having no ARB or ACE inhibitor exposures in the year prior to PC diagnosis were further restricted to those with no comorbid hypertension diagnosis or other antihypertensive medication exposures that year, the associations were stronger for ARBs after PC diagnosis (Supplemental Table S7: HR = 0.65; 95% CI: 0.47, 0.91) and for ACE inhibitors during the first 3 years of survival after PC diagnosis (Supplemental Table S8: HR = 0.72; 95% CI: 0.59, 0.89). There were no subgroups that showed survival benefits from exposure to ACE inhibitors after three years of survival. Supplemental Table S9 shows the crude and adjusted HRs for both ARBs and ACE inhibitor exposures in all patients and in each subgroup. The differences observed between the respective crude and adjusted HRs suggest that confounding was strong for some estimates, such as for the ARB HR among metastatic patients: crude HR = 1.08 vs. adjusted HR = 0.76.

## Discussion

Having analyzed over 8,158 PC case records, this is by far the largest study of PC patient survival related to post-diagnosis exposure to ARBs or ACE inhibitors. Our results suggest that these drugs are significantly associated with improved prognosis. The PC patients that received at least one ARB prescription after their PC diagnosis experienced substantial mortality risk reductions of 20% in general, but 28% among the resected PC patients, 24% among those with metastatic PC at diagnosis, and 26% among those with at least one comorbidity. ACE inhibitors were associated with improving survival in PC patients, but only during their first three years of survival in which they experienced mortality risk reductions of 13% in general and 17% among non-metastatic patients not resected – a subgroup likely representing locally advanced PC patients that, in our study, did not appear to benefit as much as others from ARB exposure. A plausible explanation for the attenuation of this association after 3 years of survival is confounding by indication. Once these PC patients had survived the cancer at least that long and were in remission, other causes of death had become more common and patients treated with ACE inhibitors likely experienced greater risk of death from non-PC causes due to their diabetes, hypertension, or other indications.

Similar to our study, at least two clinical studies and several other observational studies of renin-angiotensin system (RAS) inhibitors have shown survival benefits in PC patients independently of chemotherapy [23], in patients undergoing gemcitabine treatment [13, 24], and in patients undergoing surgical resection for PC [14]. A recent single-arm phase II clinical trial was initiated to investigate losartan’s potential for improving success in surgical tumor resection among locally advanced pancreatic ductal adenocarcinoma (PDA) patients receiving FOLFIRINOX followed by chemoradiotherapy [15]. Their findings suggest a possible benefit from losartan and also support further clinical studies, such as the currently ongoing 4-arm randomized phase II clinical trial of the effects of losartan and/or immunotherapy (nivolumab) in combination with FOLFIRINOX and stereotactic body radiotherapy (SBRT) on improving success in surgical tumor resection in patients with localized PC (NCT03563248) [16]. However, our study is the first to analyze survival following ARB and ACE inhibitors exposure in a general PC population with focused subgroup analysis of metastatic PC patients showing significantly improved survival in that particularly high-risk subpopulation of patients.

To understand how ARBs and ACE inhibitors may have affected improved survival in PC patients, consider that angiotensin II is a central hormone in the RAS that helps maintain fluid and electrolyte homeostasis throughout the body [4]. Past evidence shows that angiotensin II and the RAS are also expressed at the local tissue level and have been associated with influencing tissue angiogenesis, cellular proliferation, and apoptosis through paracrine functions [25]. Angiotensin II has two well-defined receptors that are prevalent in human tissue, the angiotensin II type 1 (AT_1_) and the angiotensin II type 2 (AT_2_) receptors. The AT_1_ receptor is primarily responsible for the cardiovascular and renal benefits seen with angiotensin inhibition, but is also a potential target for antineoplastic agents. In particular, specific inducement of the AT_1_ receptor is linked with increased cancer cell proliferation, growth, and reduced rates of apoptosis [26]. The AT_2_ receptor, however, is only expressed in adult tissue in a limited capacity and is suspected to antagonize many proposed antineoplastic functions of the AT_1_ receptor [27]. ARBs and ACE inhibitors primarily regulate the RAS through either the competitive inhibition of angiotensin-converting-enzyme responsible for producing angiotensin II or directly inhibiting the binding of angiotensin II at the AT_1_ receptor. Therefore, as ACE inhibitors prevent the systemic formation of angiotensin II, they inhibit the effect of angiotensin II on both the AT_1_ and AT_2_ receptors while ARBs, as competitive receptor antagonists, only inhibit angiotensin II at its primary binding site, the AT_1_ receptor. Initial studies identified angiotensin II as a strong mediator of VEGF expression in PDA cells due to an AT_1_ dependent pathway [6, 7]. Direct inhibition of the AT_1_ receptor with the ARB losartan was shown to reduce pancreatic tumor size in mice and rats, presumably due to suppression of VEGF-mediated angiogenesis [28, 29]. Losartan also appears to have a significant impact on PDA cell survival through stimulation of p53 directed apoptosis [30] and was shown to improve intratumoral drug delivery to PC tumors in mice [31]. In vitro studies also show that losartan, when used with nanoparticle doxorubicin, reduced tumor size and increased intratumoral distribution of doxorubicin nanoparticles likely due to inhibition of collagen I production within cells. [32]. As angiotensin II inhibition is reported to decrease the expression of various growth factors which influence the tumor–stromal interaction, exposure to ARBs and ACE inhibitors may modulate the tumor microenvironment against PC by decreasing the stromal volume and improving drug delivery to increase the efficacy of chemotherapeutics. [33]. Theories behind the antineoplastic potential of ACE inhibitors remain unsettled; multiple studies examining inhibition of the AT_2_ receptor have produced competing mechanisms. Stimulation of the AT_2_ receptor is associated with reducing PC cell growth, a benefit that disappeared with use of an AT_2_-specific antagonist [34]. However, at least one study found that AT_2_ inhibition was associated with decreased fatty acid synthase translation and decreased PC cell survival suggesting there may be alternate mechanisms for ACE inhibitors in PC [35].

There are important limitations and strengths to our claims-based retrospective cohort study. There were no records available in the database on behavioral exposures, such as smoking history, that could be important risk factors for PC and hypertension and would be useful as covariates in our models. The outpatient pharmacy records did not indicate the dose of any of the drugs prescribed. As such, we were unable to describe the dose-response relationship between post PC diagnosis ARB or ACE inhibitor exposure and survival. Moreover, we could not ascertain how well the patients adhered to their prescriptions. We recognize the inherent limitations of using an administrative healthcare database for research purposes. For instance, diagnosis and procedures coding in the database could have led to a misclassification of metastasis at diagnosis or an incomplete identification of exactly which patients had which comorbidities. We do not think that the likelihood of being coded for comorbidity was imbalanced between those who were and those who were not prescribed ACE inhibitors or ARBs in such a way that would have biased the results on their exposures and mortality away from the null (i.e., latent comorbidity having higher prevalence among those exposed to ACE/ARBs leading to stronger associations between these exposures and mortality). It is important to note, though, that we have been able to capture and adjust our models for critically important information on comorbid conditions through tracking outpatient drug prescriptions and using them as baseline and time-varying exposure covariates. Despite covariate adjustments, the comparison of ARB or ACE inhibitor exposed and unexposed PC patients could have been confounded to some degree by selection bias related to the severity or complications of their respective PC cases. Still, our careful utilization of the available covariate information and construction of time-dependent drug exposure variables and considerations for time-dependent risk parameter coefficients supports the internal validity of the results. Having comprehensive survival, outpatient prescribing, and comorbidity data on all PC cases in the entire Emilia-Romagna adult population of Italy from 2003 to 2011 is a great and perhaps unique strength of our study which supports the generalizability of our findings.

What could be the clinical implications of the study findings? The prospect of repurposed inexpensive and well-tolerated medications, such as ARBs and ACE inhibitors, having an impact on survival in PC patients is certainly intriguing from a clinical standpoint – especially considering that there are so few effective interventions available for PC patients, besides surgical resection when possible. Still, the associations we have estimated in this observational study need to be interpreted with caution as they are only a step in the process toward arriving at reliable answers to questions on clinical utility. However, it may be reasonable for PC patients requiring treatment for other indications, such as hypertension, to preferentially use ARBs or ACE inhibitors over other antihypertensives, and for PC patients already using ARBs or ACE inhibitors to weigh the potential for a survival benefit when considering deprescribing preventative treatments for chronic conditions. Given the relative strength and consistency of survival benefit associated with ARBs in our data, as compared with ACE inhibitors, it is tempting to prioritizing ARBs, such as losartan, for interventional studies in the general population of newly diagnosed PC patients. However, we believe that further observational studies and clinical trials of both classes are warranted.

## Conclusions

More prospective clinical evidence is needed for determining the benefits of ARBs and ACE inhibitors on survival in PC patients before considering treatment recommendations on the use, duration, and dosing of these drugs in the broad target population. Nevertheless, our study suggests there is hope for prolonged survival among PC patients who take ARBs or ACE inhibitors. We have shown compelling associations between reduced mortality risk and exposure to ARBs or ACE inhibitors, including analysis of how long after PC diagnosis survival benefits might be expected. Considering that these common FDA approved drugs are inexpensive to payers and present minimal increased risk of adverse events to patients, there is an urgent need for randomized clinical trials, large simple randomized trials, and pragmatic clinical trials to formally and broadly evaluate the effects of ARBs and ACE inhibitors on survival in PC patients.

## Supplementary Information

**Additional file 1.**

## Data Availability

Data for this study were retrieved from the Regional database of the Emilia-Romagna Region, provided through a collaborative agreement between the Regional Health Care and Social Agency, Regione Emilia-Romagna, Italy, the Health Care Authority, Regione Emilia- Romagna, Italy, and Thomas Jefferson University. Restrictions apply to the availability of these data, which were used under license for the current study, and so are not publicly available.

## Abbreviations

ACE: angiotensin I converting enzyme
ARB: angiotensin II receptor blocker
AT_1_: angiotensin II type 1
AT_2_: angiotensin II type 2
CI: confidence interval
FDA: United States Food and Drug Administration
HR: hazard ratio
ICD-9-CM: International Classification of Diseases, Ninth Revision, Clinical Modification
NSAID: non-steroidal anti-inflammatory drug
PC: pancreatic cancer
PDA: pancreatic ductal adenocarcinoma
RAS: renin-angiotensin system
SBRT: stereotactic body radiotherapy
VEGF: vascular endothelial growth factor
