# Supplementary material for: Angiotensin blockade therapy and survival in pancreatic cancer: a population study
Source: BMC Cancer. 2022 Feb 7;22:150. doi: 10.1186/s12885-022-09200-4 (PMC8819908; doi:10.1186/s12885-022-09200-4)
Supplement: Supplementary file 1 — Additional file 1. [file 12885_2022_9200_MOESM1_ESM.docx]

**Supplementary Appendix to** “**Angiotensin blockade therapy and survival in pancreatic cancer: A population study.**”

**Authors:** Scott W. Keith, PhD; Vittorio Maio, PharmD, MS, MSPH; Hwyda Arafat, MD, PhD; Matthew Alcusky, PharmD; Thomas Karagiannis, PharmD; Carol Rabinowitz; Harish Lavu, MD, FACS; Daniel Z. Louis, MS

**Table S1. ICD-9 diagnostic codes for identifying metastatic and non-metastatic pancreatic cancer patients in the administrative RER database.** A review of all hospital discharge records from Regione Emilia-Romagna for the period 2002 through 2011 was completed. All primary and secondary diagnosis codes were examined. An admission for cancer of the pancreas was identified as containing at least 1 of the following codes:

| 157.0 Malignant neoplasm pancreas head |
| --- |
| 157.1 Malignant neoplasm pancreas body |
| 157.2 Malignant neoplasm pancreas tail |
| 157.3 Malignant neoplasm pancreatic duct |
| 157.9 Malignant neoplasm pancreas part unspecified |

**Table S2. ICD-9-CM codes to capture lymph node involvement and metastases**.

Stage IV tumors were identified by the presence of any of the following diagnosis codes:

| 196.0 Secondary malignant neoplasm lymph node head, face neck |
| --- |
| 196.1 Secondary malignant neoplasm intrathoracic lymph nodes |
| 196.5 Secondary malignant neoplasm lymph node inguinal region, lower limb |
| 196.6 Secondary malignant neoplasm intrapelvic lymph nodes |
| 196.8 Secondary malignant neoplasm lymph nodes of multiple sites |
| 196.9 Secondary malignant neoplasm lymph nodes site unspecified |
| 197.0 Secondary malignant neoplasm of lung |
| 197.4 Secondary malignant neoplasm small intestine, including duodenum |
| 197.5 Secondary malignant neoplasm large intestine and rectum |
| 197.6 Secondary malignant neoplasm retroperitoneum and peritoneum |
| 197.7 Secondary malignant neoplasm liver, specified as secondary |
| 197.8 Secondary malignant neoplasm other digestive organs and spleen |
| 198.3 Secondary malignant neoplasm brain and spinal cord |
| 198.4 Secondary malignant neoplasm other parts of the nervous system |
| 198.5 Secondary malignant neoplasm bone and bone marrow |
| 198.6 Secondary malignant neoplasm ovary |
| 198.7 Secondary malignant neoplasm adrenal gland |
| 198.89 Secondary malignant neoplasm other specified sites |
| 199.0 Malignant neoplasm without specification of site disseminated |
| 199.1 Malignant neoplasm without specification of site other |

**Table S3.** **ICD-9 codes indicating pancreas resection.** Pancreatic cancer surgical procedures on or after the date of pancreatic cancer diagnosis were identified by the presence of any of the following codes:

| 52.51 Proximal pancreatectomy |
| --- |
| 52.52 Distal pancreatectomy |
| 52.53 Radical subtotal pancreatectomy |
| 52.59 Other partial pancreatectomy |
| 52.6 Total pancreatectomy |
| 52.7 Radical pancreaticduodenectomy |

**Table S4. A hypothetical time-dependent covariates coding example.**

| ID | Diagnosis date | Last Follow-up | Start time* | Stop time* | Dead | Quarter | ACE Rx | **ACE indicator** | ARB  Rx | **ARB indicator** |
| --- | --- | --- | --- | --- | --- | --- | --- | --- | --- | --- |
| 101 | 1/1/2005 | 6/4/2005 | . | . | . | -5 | 1 | . | 0 | . |
| 101 | 1/1/2005 | 6/4/2005 | . | . | . | -4 | 1 | . | 0 | . |
| 101 | 1/1/2005 | 6/4/2005 | . | . | . | -3 | 1 | . | 0 | . |
| 101 | 1/1/2005 | 6/4/2005 | . | . | . | -2 | 0 | . | 0 | . |
| 101 | 1/1/2005 | 6/4/2005 | . | . | . | -1 | 1 | . | 0 | . |
| 101 | 1/1/2005 | 6/4/2005 | 0 | .25 | 0 | 1 | 1 | **1** | 0 | **0** |
| 101 | 1/1/2005 | 6/4/2005 | .25 | .5 | 0 | 2 | 0 | **1** | 1 | **0** |
| 101 | 1/1/2005 | 6/4/2005 | .5 | .51 | 1 | 3 | 0 | **1** | 0 | **1** |
| 202 | 12/31/2005 | 11/1/2006 | . | . | . | -5 | 0 | . | 1 | . |
| 202 | 12/31/2005 | 11/1/2006 | . | . | . | -4 | 0 | . | 1 | . |
| 202 | 12/31/2005 | 11/1/2006 | . | . | . | -3 | 0 | . | 1 | . |
| 202 | 12/31/2005 | 11/1/2006 | . | . | . | -2 | 0 | . | 1 | . |
| 202 | 12/31/2005 | 11/1/2006 | . | . | . | -1 | 0 | . | 1 | . |
| 202 | 12/31/2005 | 11/1/2006 | 0 | .25 | 0 | 1 | 0 | **0** | 1 | **1** |
| 202 | 12/31/2005 | 11/1/2006 | .25 | .5 | 0 | 2 | 1 | **0** | 0 | **1** |
| 202 | 12/31/2005 | 11/1/2006 | .5 | .75 | 0 | 3 | 1 | **1** | 0 | **1** |
| 202 | 12/31/2005 | 11/1/2006 | .75 | 0.91 | 1 | 4 | 1 | **1** | 0 | **1** |

*Counting process style of survival time input for structuring time-dependent drug prescription exposure variables (ACE indicator and ARB indicator) every quarter-year period up to and after PC diagnosis.

**Table S5. Summary of ACE inhibitors or ARBs exposure following pancreatic cancer diagnosis.**

| **Drug Class** | **Patients Exposed** | | **Median Exposure Time (quarters)** | **1^st^, 3^rd^ quartiles (quarters)** |
| --- | --- | --- | --- | --- |
|  | **No.** | **%** |  |  |
| **ACE Inhibitors** | 2,591 | 31.8 | 2 | 1, 4 |
| **ARBs** | 1,470 | 18.0 | 2 | 1, 4 |

Abbreviations: angiotensin I converting enzyme (ACE); angiotensin II receptor blocker (ARB)

**Table S6. Complete Results from the All Patients Model**^‡^ **Summarized for ARBs and ACE inhibitors in Figures 1 & 2.**

| **Variable** | **HR** | **95% CI** | **p-value** |
| --- | --- | --- | --- |
| **Time-dependent ARBs and ACE-Inhibitors Exposures After PC diagnosis** |  |  |  |
| ARBs | **0.80** | **(0.72, 0.89)** | **0.0004** |
| ACE inhibitors (within 3 yrs of Dx) | **0.87** | **(0.80, 0.94)** | **<.0001** |
| ACE inhibitors (>3 yrs after Dx) | **1.14** | **(0.90, 1.45)** | **0.2849** |
| **Pancreatic Cancer Related** |  |  |  |
| Age/10 years at PC | 1.26 | (1.22, 1.29) | <.0001 |
| Age/10 years squared | 1.01 | (1.00, 1.03) | 0.1282 |
| Metastatic | 2.33 | (2.21, 2.46) | <.0001 |
| Pancreas resection | 0.53 | (0.49, 0.57) | <.0001 |
| Chemotherapy | 0.96 | (0.91, 1.02) | 0.2130 |
| Radiotherapy | 1.24 | (1.14, 1.34) | <.0001 |
| **Demographics** |  |  |  |
| Sex (male) | 1.12 | (1.06, 1.17) | <.0001 |
| Diagnosis calendar time (/yr) | 0.99 | (0.98, 1.00) | 0.0046 |
| Geography |  |  |  |
| Hill vs. Plain | 1.08 | (1.02, 1.14) | 0.0049 |
| Mountain vs. Plain | 1.14 | (1.03, 1.27) | 0.0125 |
| **Comorbidity** |  |  |  |
| Elixhauser comorbidities score[^19^](#_ENREF_19) | 0.98 | (0.94, 1.03) | 0.3962 |
| Most Prevalent Elixhauser Comorbidities |  |  |  |
| Deficiency Anemias | 1.02 | (0.89, 1.18) | 0.7814 |
| Chronic Blood Loss | 0.90 | (0.73, 1.10) | 0.3048 |
| Chronic Pulmonary Disease | 1.01 | (0.91, 1.13) | 0.8038 |
| Pulmonary Circulation Disease | 1.37 | (1.14, 1.65) | 0.0006 |
| Peripheral Vascular Disease | 0.98 | (0.84, 1.14) | 0.7399 |
| Depression | 1.05 | (0.87, 1.28) | 0.5945 |
| Hypothyroidism | 0.96 | (0.81, 1.15) | 0.6868 |
| Liver Disease | 0.97 | (0.84, 1.12) | 0.6810 |
| Neurological Disorders | 1.02 | (0.87, 1.20) | 0.8087 |
| **Functional Status** |  |  |  |
| Home health care or O_2_ prescription in year prior to PC diagnosis | 1.17 | (1.07, 1.27) | 0.0004 |
| Discharged to nursing home | 1.81 | (1.65, 1.99) | <.0001 |
| **Other Time-Dependent Drug Exposures After PC diagnosis** |  |  |  |
| Aspirin | 1.01 | (0.94, 1.09) | 0.7162 |
| Alpha blockers | 0.93 | (0.79, 1.09) | 0.3628 |
| Beta blockers | 0.98 | (0.90, 1.07) | 0.6428 |
| Calcium channel blockers | 1.00 | (0.92, 1.09) | 0.9553 |
| Diuretics | 1.67 | (1.57, 1.78) | <.0001 |
| NSAIDs | 0.98 | (0.59, 1.63) | 0.9331 |
| Statins | 0.89 | (0.80, 0.98) | 0.0225 |
| Metformin | 0.92 | (0.83, 1.02) | 0.1085 |
| Other diabetes medications | 1.32 | (1.22, 1.42) | <.0001 |
| **Other Drug Exposures in year prior to PC diagnosis** |  |  |  |
| ACE inhibitors | 1.08 | (1.00, 1.16) | 0.0504 |
| ARBs | 1.19 | (1.07, 1.32) | 0.0013 |
| Aspirin | 0.96 | (0.89, 1.03) | 0.2247 |
| Alpha blockers | 0.93 | (0.80, 1.09) | 0.3574 |
| Beta blockers | 1.01 | (0.92, 1.11) | 0.8319 |
| Calcium channel blockers | 0.97 | (0.89, 1.06) | 0.4760 |
| Diuretics | 0.72 | (0.67, 0.78) | <.0001 |
| NSAIDs | 1.00 | (0.86, 1.16) | 0.9920 |
| Statins | 1.08 | (0.99, 1.19) | 0.0987 |
| Metformin | 1.01 | (0.91, 1.13) | 0.8376 |
| Other diabetes medications | 0.90 | (0.82, 0.98) | 0.0190 |

Abbreviations: angiotensin I converting enzyme (ACE); angiotensin II receptor blocker (ARB); antidiabetic medications other than Metformin (Other DM Rx); hypertension prescriptions (HTN Rx); pancreatic cancer (PC)

^‡^ Estimated by Cox models of a time-dependent ARB prescription exposure indicator and two time-dependent ACE inhibitor prescription exposure indicators (one for exposure within 3 years of diagnosis, another for exposure after 3 years of survival) adjusted for covariates including the exposure in the year prior to diagnosis to at least one prescription filled for ARBs, ACE inhibitors, aspirin, alpha blockers, beta blockers, Ca channel blockers, diuretics, NSAIDs, statins, metformin, and other diabetes medications, respectively; a set of post PC diagnosis time-dependent exposure covariates for the latter 9 drug exposures listed, chemotherapy, radiation therapy, and pancreas resection; sex; age (and age^2^); Elixhauser comorbidity score; indicator variables for some of the most prevalent comorbidities listed in Table 1; functional status variables (home health care or oxygen prescription in year prior to PC diagnosis and discharge to nursing home); geographic location of residence indicators (hill vs. plain and mountain vs. plain); and distant metastatic PC at diagnosis.

**Table S7. Subgroup modeling results for ARBs exposure after PC diagnosis among those having no ARB or ACE inhibitor exposures in the year prior to PC diagnosis when further restricted to those with and those without antihypertensive medication exposure or hypertension diagnosis code that year.**

| **Subgroup** | **ARB HR** | **95% CI** |  |
| --- | --- | --- | --- |
| **No prior ACE or ARB** | 0.77 | (0.62, 0.95) |  |
| **Hypertensives*** | 0.94 | (0.69, 1.26) |  |
| **No Hypertensives** | 0.65 | (0.47, 0.91) |  |
| *No prior ACE or ARB prescriptions, but have had a hypertension diagnostic code or prescription for anti-hypertensive medication (i.e., beta blocker, alpha blocker, calcium channel blocker, or diuretics) in the year prior to PC diagnosis. | | | |

**Table S8. Subgroup modeling results for ACE inhibitors exposure after PC diagnosis among those having no ARB or ACE inhibitor exposures in the year prior to PC diagnosis when further restricted to those with and those without antihypertensive medication exposure or hypertension diagnosis code that year.**

| **Survival Period** | **Subgroup** | **ACE HR** | **95% CI** |  |
| --- | --- | --- | --- | --- |
| **Up to 3 years** |  |  |  |  |
|  | **No prior ACE or ARB** | 0.78 | (0.67, 0.89) |  |
|  | **Hypertensives*** | 0.85 | (0.69, 1.04) |  |
|  | **No Hypertensives** | 0.72 | (0.59, 0.89) |  |
| **After 3 years** |  |  |  |  |
|  | **No prior ACE or ARB** | 1.10 | (0.79, 1.55) |  |
|  | **Hypertensives*** | 1.24 | (0.74, 2.07) |  |
|  | **No Hypertensives** | 0.93 | (0.57, 1.50) |  |
| *No prior ACE or ARB prescriptions, but have had a hypertension diagnostic code or prescription for anti-hypertensive medication (i.e., beta blocker, alpha blocker, calcium channel blocker, or diuretics) in the year prior to PC diagnosis. | | | | |

**Table S9. Death totals, crude hazard ratios, and adjusted hazard ratios by pancreatic cancer patient subgroups.**

|  |  |  | **ARB** | **ARB** | **ACE (up to 3 years)** | **ACE (up to 3 years)** | **ACE (after 3 years)** | **ACE (after 3 years)** |
| --- | --- | --- | --- | --- | --- | --- | --- | --- |
| **Patient Subgroup** | **Sample Size** | **Deaths** | **Crude HR**  **(95% CI)** | **Adjusted HR**  **(95% CI)** | **Crude HR**  **(95% CI)** | **Adjusted HR**  **(95% CI)** | **Crude HR**  **(95% CI)** | **Adjusted HR**  **(95% CI)** |
| All patients | 8154 | 7027 (86.1%) | 0.95  (0.89, 1.01) | 0.80  (0.72, 0.89) | 1.05  (0.99, 1.11) | 0.87  (0.80, 0.94) | 1.42  (1.12, 1.79) | 1.14  (0.90, 1.45) |
| Metastatic patients | 2955 | 2857 (96.7%) | 1.08  (0.97, 1.21) | 0.76  (0.64, 0.90) | 1.12  (1.03, 1.21) | 0.88  (0.77, 1.00) | 1.67  (0.69, 4.05) | 1.67  (0.64, 4.09) |
| Resected patients | 1613 | 1032 (64.0%) | 0.91  (0.78, 1.07) | 0.72  (0.55, 0.96) | 1.06  (0.92, 1.22) | 0.95  (0.76, 1.18) | 1.19  (0.84, 1.69) | 1.11  (0.76, 1.61) |
| No mets & Not resected | 3590 | 3138 (87.4%) | 0.94  (0.86, 1.03) | 0.89  (0.76, 1.04) | 0.97  (0.90, 1.05) | 0.83  (0.74, 0.93) | 1.51  (1.07, 2.14) | 1.23  (0.86, 1.76) |
| No prior ACE or ARB | 4377 | 3737 (85.4%) | 0.89  (0.72, 1.10) | 0.77  (0.62, 0.95) | 0.96  (0.84, 1.10) | 0.78  (0.67, 0.89) | 1.39  (1.00, 1.94) | 1.10  (0.79, 1.55) |
| Prior ACE or ARB | 3781 | 3290 (87.0%) | 0.83  (0.76, 0.91) | 0.82  (0.72, 0.93) | 0.91  (0.83, 0.99) | 0.91  (0.82, 1.01) | 1.45  (0.94, 2.21) | 1.32  (0.86, 2.02) |

**Figure S1. Covariate-adjusted time-dependent mortality hazard ratios associated with time-dependent exposures to ARBs (panel A) or ACE inhibitors (panel B) after pancreatic cancer diagnosis.** These results suggest the association between ARBs and mortality in PC patients does not depend on how long the patients have survived since diagnosis. However, the association for ACE inhibitors appears to attenuate substantially after 3 years of survival.

| **A.** |
| --- |
| **B.** |
